# Real-Time RT-PCR Allelic Discrimination Assay for Detection of N501Y Mutation in the Spike Protein of SARS-CoV-2 Associated with B.1.1.7 Variant of Concern

**DOI:** 10.1128/spectrum.00681-21

**Published:** 2022-02-16

**Authors:** Mariana Abdulnoor, AliReza Eshaghi, Stephen J. Perusini, George Broukhanski, Antoine Corbeil, Kirby Cronin, Nahuel Fittipaldi, Jessica D. Forbes, Jennifer L. Guthrie, Julianne V. Kus, Ye Li, Anna Majury, Gustavo V. Mallo, Tony Mazzulli, Roberto G. Melano, Romy Olsha, Ashleigh Sullivan, Vanessa Tran, Samir N. Patel, Vanessa G. Allen, Jonathan B. Gubbay

**Affiliations:** a Public Health Ontario, Toronto, Ontario, Canada; b Department of Laboratory Medicine and Pathobiology, University of Toronto, Toronto, Ontario, Canada; c Department of Microbiology, Sinai Health/University Health Network, Toronto, Ontario, Canada; d Dalla Lana School of Public Health, University of Toronto, Toronto, Ontario, Canada; e Division of Infectious Diseases, Department of Paediatrics, The Hospital for Sick Children, Toronto, Ontario, Canada; Children's Hospital Los Angeles, University of Southern California

**Keywords:** COVID-19, real-time RT-PCR, SARS-CoV-2, VOC

## Abstract

The N501Y amino acid mutation caused by a single point substitution A23063T in the spike gene of severe acute respiratory syndrome coronavirus 2 (SARS-CoV-2) is possessed by three variants of concern (VOCs), B.1.1.7, B.1.351, and P.1. A rapid screening tool using this mutation is important for surveillance during the coronavirus disease 2019 (COVID-19) pandemic. We developed and validated a single nucleotide polymorphism real-time reverse transcription PCR assay using allelic discrimination of the spike gene N501Y mutation to screen for potential variants of concern and differentiate them from SARS-CoV-2 lineages without the N501Y mutation. A total of 160 clinical specimens positive for SARS-CoV-2 were characterized as mutant (N501Y) or N501 wild type by Sanger sequencing and were subsequently tested with the N501Y single nucleotide polymorphism real-time reverse transcriptase PCR assay. Our assay, compared to Sanger sequencing for single nucleotide polymorphism detection, demonstrated positive percent agreement of 100% for all 57 specimens displaying the N501Y mutation, which were confirmed by Sanger sequencing to be typed as A23063T, including one specimen with mixed signal for wild type and mutant. Negative percent agreement was 100% in all 103 specimens typed as N501 wild type, with A23063 identified as wild type by Sanger sequencing. The identification of circulating SARS-CoV-2 lineages carrying an N501Y mutation is critical for surveillance purposes. Current identification methods rely primarily on Sanger sequencing or whole-genome sequencing, which are time consuming, labor intensive, and costly. The assay described herein is an efficient tool for high-volume specimen screening for SARS-CoV-2 VOCs and for selecting specimens for confirmatory Sanger or whole-genome sequencing.

**IMPORTANCE** During the coronavirus disease 2019 (COVID-19) pandemic, several variants of concern (VOCs) have been detected, for example, B.1.1.7, B.1.351, P.1, and B.1.617.2. The VOCs pose a threat to public health efforts to control the spread of the virus. As such, surveillance and monitoring of these VOCs is of the utmost importance. Our real-time RT-PCR assay helps with surveillance by providing an easy method to quickly survey SARS-CoV-2 specimens for VOCs carrying the N501Y single nucleotide polymorphism (SNP). Samples that test positive for the N501Y mutation in the spike gene with our assay can be sequenced to identify the lineage. Thus, our assay helps to focus surveillance efforts and decrease turnaround times.

## INTRODUCTION

Severe acute respiratory syndrome coronavirus 2 (SARS-CoV-2) is the viral etiology of the coronavirus disease 2019 (COVID-19) pandemic. Several SARS-CoV-2 variants of concern (VOCs) have been identified, which are associated with increased transmissibility, increased virulence, changes in clinical disease presentation, and decreased effectiveness of public health measures, diagnostics, vaccines, and therapeutics (1). Throughout the pandemic, four novel VOCs of SARS-CoV-2 emerged independently (2–5).

The VOC first detected in the United Kingdom represents the B.1.1.7 lineage (also known as 20I/501Y.V1, VOC202012/010, and Alpha) and contains three key mutations in the spike (S) gene: 69 to 70 deletion, N501Y, and P681H. The E484K mutation has been observed in some B.1.1.7 genome sequences (6). B.1.1.7 is associated with increased transmissibility of 50 to 70% (7) and increased disease severity and risk of death (8). Cases of B.1.1.7 lineage have been found in 114 countries (https://cov-lineages.org/global_report_B.1.1.7.html). In Ontario, Canada, 2,165 cases of B.1.1.7 have been confirmed as of 5 April 2021 (9).

A second SARS-CoV-2 VOC representing the B.1.351 lineage (also known as 20H/501Y.V2, VOC-202012/02, and Beta) emerged in October 2020 in South Africa (3). This VOC has multiple mutations in the receptor-binding domain (RBD) of the S protein, including K417N, E484K, and N501Y. The B.1.351 lineage is associated with increased transmissibility (10) and immune evasion (11, 12). It is unknown how this VOC influences disease severity, hospitalizations, and deaths. B.1.351 has been detected in 68 countries (https://cov-lineages.org/global_report_B.1.351.html), including Canada, where in Ontario, there are 71 confirmed cases as of 5 April 2021 (9).

A third VOC is the P.1 lineage (also known as B.1.1.28.1, 20J/501Y.V3, VOC-202101/02, and Gamma), first detected in Japan from travelers returning from Brazil (13). This VOC shares the S gene N501Y single nucleotide polymorphism (SNP) with B.1.1.7 and the E484K SNP with B.1.351; however, all three lineages arose independently (4). The P.1 lineage may have higher inherent transmissibility than the previous lineages (13). This lineage is associated with antigenic escape (12). The P.1 lineage has been identified in 36 countries (https://cov-lineages.org/global_report_P.1.html), including Canada, where there are 106 confirmed cases in Ontario as of 5 April 2021 (9).

The fourth VOC is the B.1.617.2 lineage (also known as 21A/S:478K and Delta), which was first detected in India in late 2020. This lineage is characterized by several S gene SNPs, including L452R, T478K, E484Q, D614G, and P681R (5). This VOC was shown to increase transmissibility (14) and reduce susceptibility to antibody neutralization from convalescent patients and sera from vaccinated individuals (15). The B.1.617.2 lineage has been identified in 131 countries (https://cov-lineages.org/global_report_B.1.617.2.html), including Canada.

These SARS-CoV-2 VOCs will likely hamper public health efforts to contain the spread of the virus. Current SARS-CoV-2 vaccines have reduced overall vaccine effectiveness (VE) for preventing infection (including asymptomatic infection), but VE against severe disease (i.e., hospitalization and death) is less affected and remains excellent (16, 17). Adequate surveillance is important to control the spread of these lineages. Current methods rely on genome sequencing, either by Sanger sequencing or whole-genome sequencing (WGS). These approaches, although of high accuracy, are labor intensive and are not amenable to rapid turnaround times, as they require extensive laboratory processing and bioinformatic analysis. They usually take several days to complete; therefore, testing capacity is quickly outpaced as the number of specimens to be analyzed increases with transmission.

The use of real-time reverse transcription PCR (rRT-PCR) to detect SNPs of relevance would facilitate rapid preliminary identification of potential VOCs and initiation of public health measures. Herein, we report the validation of a laboratory-developed SNP rRT-PCR protocol to detect the presence of N501Y, which can act as a screening tool for three circulating VOCs in Ontario (B.1.1.7, B.1.351, and P.1), as they all share this SNP. Specimens with the N501Y mutation detected are subsequently submitted for WGS to confirm the presence of a VOC and identify the SARS-CoV-2 VOC lineage.

## RESULTS

### Accuracy—positive percent agreement and negative percent agreement for N501Y detection.

To analyze the performance of the N501Y SNP assay, a panel of 160 specimens, including 57 specimens with N501Y detected by Sanger sequencing and 103 wild-type N501 specimens, were tested. One of the 57 N501Y-positive specimens contained a mixed result on Sanger sequencing, indicating the presence of both wild-type N501 and mutant N501Y. All specimens with a known A23063T substitution in the Sanger chromatogram produced a positive signal for N501Y with the rRT-PCR SNP assay (57/57, 100%) and were confirmed to be of the B.1.1.7 lineage by Sanger sequencing analysis. All specimens with known A23063 wild-type characterization by Sanger sequencing were positive for the wild-type N501 target with the rRT-PCR SNP assay (103/103, 100%). (Table 1).

**TABLE 1:** Summary of accuracy testing data for PHO Laboratory SARS-CoV-2 N501Y SNP rRT-PCR assay

SNP detection	Sanger mutant (N501Y)^*a*^	Sanger wild type (N501)	Total
SNP (N501Y) detected	57	0	57
SNP (N501) not detected	0	103	103
Total	57	103	160

a There was one specimen with a mixed population of wild-type and mutant, this specimen was counted as positive for N501Y.

**FIG 1:**
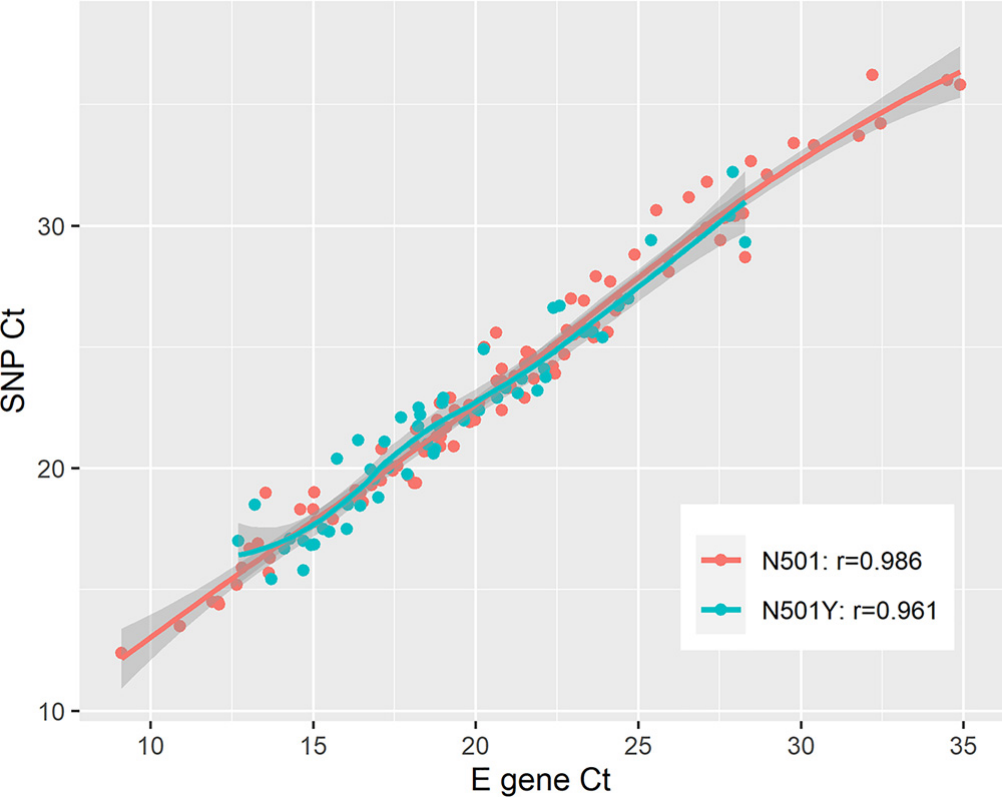
Scatterplot of N501 and N501Y SNP rRT-PCR target *C_T_* values against E gene *C_T_* values showing strong linear correlation. Regression lines were produced using simple linear regression. The N501 target is shown in red, and the N501Y target is shown in blue; N501, *r* = 0.99; N501Y, *r* = 0.96.

**FIG 2:**
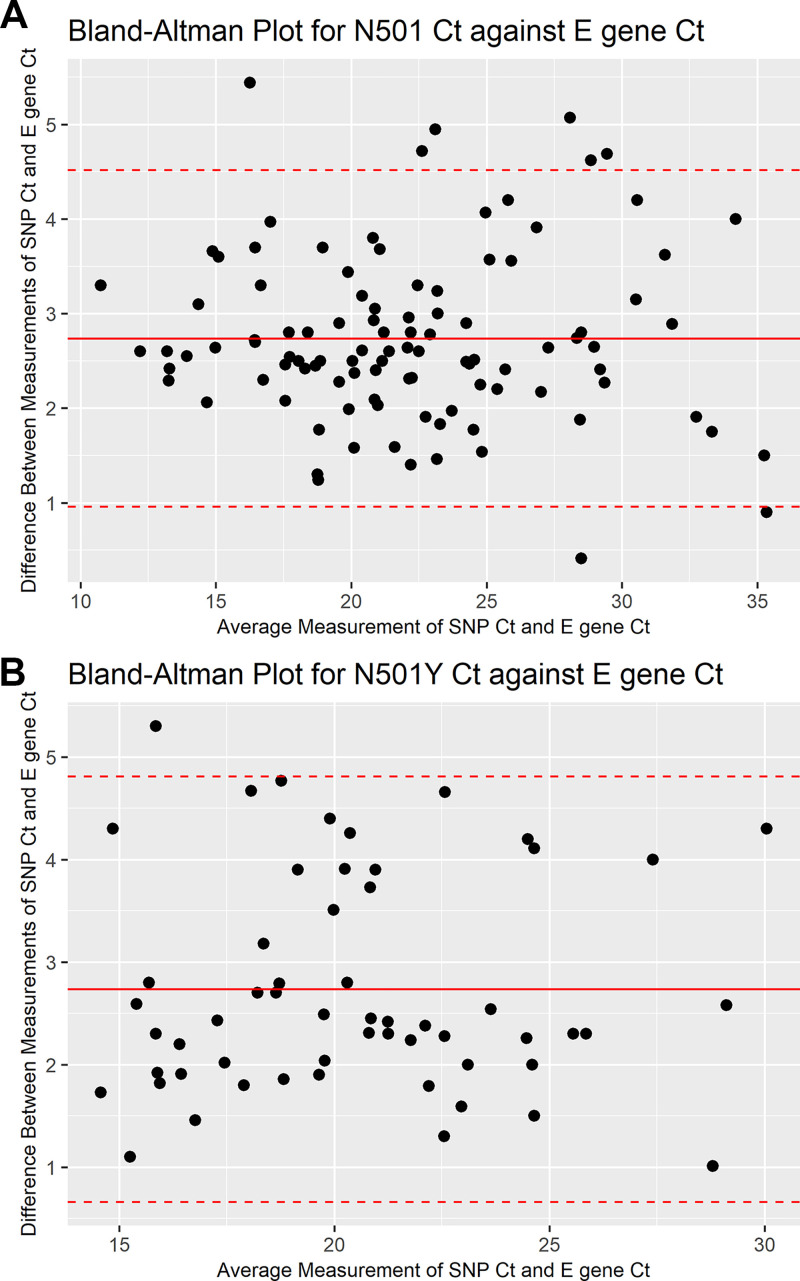
Bland-Altman plot for rRT-PCR SNP assay target *C_T_* values against E gene *C_T_* values. (A) N501 target *C_T_* values against E gene *C_T_* values. (B) N501Y target *C_T_* values against E gene *C_T_* values.

The mean envelope (E) gene and N501Y SNP rRT-PCR assay cycle threshold (*C_T_*) values of all VOC specimens in this study were 19.15 (standard deviation [SD] of 3.76) and 21.88 (SD of 3.80), respectively. The mean *C_T_* values of E gene and N501 SNP rRT-PCR for wild-type specimens were 20.87 (SD of 5.38) and 23.75 (SD of 5.52), respectively. The E gene *C_T_* values are strongly correlated with *C_T_* values of both SNP rRT-PCR assay targets, with an *r* value of 0.99 for the N501 target, which is slightly higher than the N501Y target with an *r* value of 0.96 (Fig. 1). The *C_T_* values of the N501 target are more clustered around the mean, demonstrating more agreement with the E gene *C_T_* than with the N501Y target (Fig. 2).

### Comparison of two primer and probe sets.

Our N501Y SNP assay was validated using primer and probe sets from two vendors, LGC, Biosearch Technologies (Middlesex, UK) and Thermo Fisher Scientific (MA, USA). An independent set of 87 specimens, including 14 mutants, 72 wild type, and one mixed population, were tested (Table 2). We observed comparable results for both primer and probe sets, as there were no meaningful differences in average *C_T_* values or number of N501Y specimens detected.

**TABLE 2:** Results of validation experiments with primer and probe sets from two vendors, LGC Biosearch Technologies and Thermo Fisher Scientific

Cycle threshold (CT) value	LGC Biosearch Technologies		Thermo Fisher Scientific	
	Wild-type (*n* = 72)	Mutant (*n* = 14)	Wild-type (*n* = 72)	Mutant (*n* = 14)
Mean *C_T_* value (SD)^*a*^	27.44 (6.86)	22.11 (6.92)	27.18 (6.80)	22.75 (7.14)
Median *C_T_* value (IQR)^*b*^	27.43 (17.33)	21.41 (17.15)	27.07 (18.63)	21.82 (17.36)

a SD, standard deviation.

b IQR, interquartile range.

### Analytical sensitivity (95% limit of detection).

The 95% limit of detection (LOD) for the N501Y target was determined to be 5.67 (95% confidence interval [95% CI] of 1.98 to 16.24) copies/reaction, which represents 226.71 (95% CI of 79.15 to 649.33) copies/mL of primary specimen. The 95% LOD for the N501 target was determined to be 5.30 (95% CI of 2.23 to 12.62) copies/reaction, which represents 212 (95% CI of 93.23 to 504.15) copies/mL of primary specimen.

### Precision—intrarun repeatability and interrun and interlaboratory reproducibility.

Excellent intrarun repeatability and interrun reproducibility was documented down to 15 copies/reaction or 599.4 copies/mL of primary specimen for the N501Y specimens and down to 25 copies/reaction or 999 copies/mL of primary specimen for the N501 wild-type specimens. Percent agreement among all replicates tested was 100% for intrarun repeatability and 100% for interrun reproducibility (Tables S2 to S5 in the supplemental material).

The analysis of interlaboratory reproducibility was done with replicates of a panel of nine N501Y samples, five wild-type samples, one mixed N501Y/N501 sample, and five negative samples. Among the six runs obtained from six laboratories, the results showed 100% agreement, with a mean *C_T_* of 28.02 (SD of 1.25; coefficient of variation of 4.46%) for mutant specimens and a mean *C_T_* of 26.18 (SD of 1.19; coefficient of variation of 4.56%) for wild-type specimens (Tables S6 and S7).

### Provincial point prevalence study.

To understand the provincial prevalence of VOCs in Ontario, 2,570 SARS-CoV-2-positive specimens reported on 20 January 2021 across the province were sent for screening using our rRT-PCR N501Y SNP assay, and 113 (4.4%) had a N501Y mutation detected. Eighty-eight specimens had been confirmed by WGS to have the N501Y SNP; 87 specimens were B.1.1.7 and 1 specimen was B.1.351. Additional results are available at Public Health Ontario’s (PHO’s) website (18).

Our rRT-PCR N501Y SNP assay has been instituted for surveillance testing across Ontario, allowing notification to public health at least 1 to 7 days earlier than relying on Sanger sequencing or WGS. On 11 March 2021, 1,138 specimens were screened with our N501Y rRT-PCR assay. Of 899 that were N501Y positive using our assay, 897 (99.8%) specimens were confirmed to have the N501Y SNP through WGS. Of the 239 specimens that were screened as N501 wild type using our assay, 238 (99.6%) were confirmed to be N501 wild type through WGS (data extracted from the PHO laboratory information system). Using this assay, we have documented a rapid rise in VOC cases, with roughly 60% of cases as of 5 April 2021 identified as a VOC (9).

## DISCUSSION

Here, we describe the validation and implementation of a N501Y SNP rRT-PCR assay to provide rapid screening of SARS-CoV-2-positive specimens for a subset of VOCs. As of 5 April 2021, three VOCs (B1.1.7, B.1.351, and P.1) were circulating in Ontario, and surveillance of these VOCs was of considerable public health importance. By 29 August 2021, the VOC B.1.617.2, first discovered in India, was the most common SARS-CoV-2 circulating in Ontario, representing >90% of specimens undergoing WGS as part of Ontario’s SARS-CoV-2 genomic surveillance purposes (19). The World Health Organization recommends VOC surveillance to help control the spread of VOCs (1). During the early response to VOCs, positive SARS-COV-2 specimens were screened for the N501Y SNP using the described rRT-PCR assay and then potentially submitted for WGS to confirm VOC identification and determine the VOC lineage. There were no adverse events from performing the N501Y SNP rRT-PCR assay on study specimens, as individuals had already been classified as having the mutation or not based on previous sequencing. Our assay is efficient and clinically validated with a high degree of positive percent agreement (PPA) and negative percent agreement (NPA) for detection of the N501Y SNP compared with Sanger sequencing. Our N501Y rRT-PCR assay has been implemented in the province for screening for VOCs. When first implemented, specimens positive for SARS-CoV-2 were screened for N501Y and then sequenced with WGS to determine the lineage if N501Y mutation was detected.

Sanger sequencing and WGS of VOCs require longer turnaround times and are expensive, and some regions may not have access to sequencing facilities or the ability to scale-up their sequencing capabilities. A two-factor screening process for B.1.1.7 detection has been employed in France to aid with surveillance efforts (20). The protocol detects the 69 to 70 deletion in the S gene of B.1.1.7, as this deletion causes S gene target failure (SGTF) in certain assays (e.g., Thermo Fisher’s TaqPath COVID-19 combo kit). Once SGTF is detected, specimens are sequenced by WGS. SGTF has been used as a proxy for the B.1.1.7 VOC (7). This method is limited to detecting VOCs carrying the 69 to 70 deletion. A multiplex rRT-PCR assay to screen for VOCs was published by Vogel et al. that targets the deletion of amino acids 3675 to 3677 in the ORF1a gene as well as the deletion of amino acids 69 to 70 in the S gene (21). The ORF1a deletion of amino acids 3675 to 3677 is present in B.1.17, B.1.351, and P.1, and the deletion of amino acids 69 to 70 in the S gene is used to differentiate B.1.17 from B.1351 and P.1. However, the ORF1a deletion is not found in all B.1.351 VOCs, as there is a monophyletic clade that does not contain this deletion. Another group published an rRT-PCR assay to detect only the N501Y SNP and requires a melting curve to confirm the specimens as wild type if the test is negative (22). Our procedure differs, as it can detect both the wild type and the SNP, avoiding the additional step of analyzing melting curves and thus providing easier interpretation of the results. In another assay, the RT-PCR multiplex targets include L452R, a SNP in the S gene that is characteristic of the B.1.617.2 (Delta) variant (23). Having this target allows the assay to detect B.1.617.2, an important VOC, and N501Y-containing VOCs, such as B.1.1.7, P.1, and B.1.351. However, it is not clear from the study whether the assay was validated, as the authors do not provide any validation data.

Because our assay only detects the N501Y SNP, new emerging lineages that are of concern that do not carry this SNP, such as B.1.525, and B.1.617.2, which has rapidly become the predominant strain in most countries, including Canada, will be missed (https://cov-lineages.org/global_report_B.1.617.2.html, https://cov-lineages.org/global_report_B.1.525.html). On 29 August 2021, 569 cases of B.1.617.2 were reported in Ontario, Canada, while there was only one case of B.1.1.7 and no cases of P.1 or B.1.351 (19). Additionally, non-VOC lineages carrying the N501Y SNP will be detected and flagged as VOCs incorrectly. The assay is also unable to differentiate between VOCs that share the N501Y SNP; therefore, an additional sequencing step is required to delineate the lineage. Other SNP assays have been developed for important S gene SNPs that characterize VOCs, including E484K (P.1, B.1.351), K417N/T (P.1, B.1.351), and L452R (B.1.617.2) (22). However, as new variants emerge, the combination of SNPs that define each variant will change, and RT-PCR targets will need to be updated to match what is circulating. Even with additional targets, definitive lineage identification is not possible based on SNP assays alone and requires sequencing. Our validation was conducted with specimens characterized as B.1.1.7, but when implemented as a screening tool, we were able to also detect B.1.351 and P.1 VOCs. Our validation data set contained a small sample size of 71 N501Y-positive specimens. By only screening specimens with an E gene *C_T_* value of ≤30, a selection bias is introduced as specimens with *C_T_* values of ≤30 arise from patients with higher viral loads. We did not conduct analytical specificity tests with a cross-reactivity panel due to limited availability of SARS-CoV-2 VOC strains. In our analysis, we did not assess baseline demographics, clinical characteristics of the patients, and severity of disease, as limited information is provided to the PHO Laboratory, which provides testing as a reference laboratory. Our assay is not intended for diagnostic purposes but rather as a screening tool to aid with faster identification of VOCs through differentiation of N501 and N501Y.

Our assay is a quick and simple tool that can be applied to screening SARS-CoV-2-positive specimens from priority groups at greater risk for VOC infection, such as international travelers, suspected SARS-CoV-2 reinfection, infection after COVID-19 vaccination, and outbreaks. Ontario implemented screening of all SARS-CoV-2-positive specimens, which can be in excess of 1,000 specimens per day, and the N501Y SNP rRT-PCR assay has allowed us to screen a high volume of specimens. Due to the high positive percent agreement, any specimen that is N501Y positive can be presumed to be a VOC while awaiting characterization by WGS. Early, high-throughput screening with PHO Laboratory’s N501Y screening assay provided the possibility of enhanced public health measures to be put in place (e.g., more expanded contact tracing) in an attempt to limit the spread of VOCs in the community during the early phase of emergence of B.1.1.7.

## MATERIALS AND METHODS

### Clinical specimens.

Public Health Ontario (PHO) Laboratory is the reference microbiology laboratory for the province of Ontario, Canada. As part of the response to detect VOCs in Ontario, PHO Laboratory developed indications for VOC screening, and at least one of the following criteria must be met: (i) SARS-CoV-2 infection (symptomatic or asymptomatic) during international travel (including the United States) or within 14 days of entry to Canada, (ii) SARS-CoV-2-positive contacts of recent international travelers, (iii) SARS-CoV-2-positive contacts of cases with confirmed SARS-CoV-2 VOC infection, (iv) suspected reinfection with SARS-CoV-2, (v) multitarget PCR assay (e.g., Thermo Fisher [TaqPath] with S gene drop out [S gene negative] and other gene target[s] positive with a *C_T_* value of <30), (vi) severe acute COVID-19 (i.e., requiring intensive care unit [ICU] admission or ICU level of care) in individuals less than 50 years old without significant comorbidities, (vii) vaccinated individuals with a subsequent laboratory-confirmed SARS-CoV-2 infection, and (viii) known or suspected super-spreading events.

Clinical specimens that met these criteria with a *C_T_* value of ≤30 underwent Sanger sequencing of a 698-bp (nucleotide positions 22516 to 23214) S gene fragment, which includes the locations of key receptor-binding domain (RBD) mutations (K417N/T, E484K, and N501Y). Specimens with mutations detected underwent further sequencing to confirm if a VOC was present. In addition, a subset of all SARS-CoV-2-positive specimens underwent WGS as part of the provincial genomic surveillance program, which included VOC screening.

A set of 160 SARS-CoV-2-positive clinical specimens collected between 20 November 2020 and 20 January 2021 submitted to PHO Laboratory from across the province that had undergone VOC screening by partial S gene sequencing, as described above, were used in the optimization and validation of the assay. The study set consisted of all SARS-CoV-2-positive specimens with N501Y available at PHO Laboratory in addition to a convenience sample of specimens wild type at position 501 (N501). Assay operators were not blinded to the prior Sanger sequencing results. Clinical information was unavailable for these specimens. All specimens were also tested using a laboratory-developed test targeting the envelope (E) gene, which was validated for clinical testing at PHO Laboratory (24). Specimen types included nasopharyngeal swabs, throat swabs, and bronchoalveolar lavage fluid that were collected in a range of validated specimen collection kits supplied to Ontario’s COVID-19 laboratory testing network (25). Collection kits varied in volumes of 2 to 3 mL of transport medium. Specimens were stored up to 72 h at 4°C, after which they were kept frozen at −80°C.

### rRT-PCR design and optimization.

Primer and probe sequences were selected using an alignment of spike nucleotide sequences of a wild-type SARS-CoV2 reference genome (GenBank accession MN908947.3) and a representative of the VOC B.1.1.7 lineage (Global Initiative on Sharing Avian Influenza Data [GISAID] accession EPI_ISL_601443). Selected primer and probe sequences were aligned to 9,000 gene sequences downloaded from GISAID with a submission date between 1 December 2020 and 6 December 2020 to ensure that they were targeting a conserved region. In addition, a BLAST search of the primer and probe sequences against the whole genome of the reference sequence (MN908947.3) was conducted to ensure the absence of homology to other parts of the SARS-CoV-2 genome. Primers and probes selected for the detection of SNP A23063T are reported in Table S1 in the supplemental material. Primers and probes were synthesized by LGC, Biosearch Technologies (Middlesex, UK). Optimization was performed using previously characterized wild-type and B.1.1.7 specimens. No B.1.351 or P.1 lineage SARS-CoV-2 specimens were detected in Ontario at the time of assay development. Optimization involved using different reverse and forward primer and probe designs to amplify the region of concern as well as titration of the primers and probes and various thermal cycler parameters (e.g., altering cycling temperatures and times). Each assay was optimized in singleplex format followed by final optimization of the multiplex assay. Once optimized, the specimens used in optimization were included in the validation data set.

An additional smaller validation using an independent specimen set was conducted using primers and probes from a second vendor, Thermo Fisher Scientific (MA, USA), to allow redundancy of reagent suppliers.

### Total nucleic acid extraction.

Specimens underwent nucleic acid extraction using either PerkinElmer chemagic 360 automated systems (PerkinElmer, MA, USA) (input volume of 300 μL; eluate volume of 60 μL) or the MGISP-960RS platform (MGI Tech Co., Guangdong, China) (input volume of 180 μL; eluate volume of 45 μL) according to the manufacturer’s recommendations. The same nucleic acid eluates were used for performing both N501Y SNP rRT-PCR and Sanger sequencing.

### N501Y SNP rRT-PCR assay.

The PCR amplification was performed using the TaqPath 1-step multiplex master mix on the Applied Biosystems QuantStudio 5 real-time PCR system (Thermo Fisher Scientific, MA, USA).

A total volume of 10 μL of reaction mix contained 2.5 μL of 4× TaqPath 1-step multiplex master mix, 1.0 μL of primer mix, 1.0 μL of probe mix, 2.5 μL of RNase-free water, and 3 μL of viral RNA extract. The default thermocycling profile suggested for 4× TaqPath 1-step multiplex master mix was used, including 20 min at 25°C, 10 min at 53°C, 2 min at 95°C, and 45 cycles of 95°C for 3 s then 60°C for 30 s. The controls used in each run were an extraction negative control that consisted of nuclease-free water processed along with SARS-CoV-2 specimens during the extraction process, a PCR negative control that consisted of nuclease-free water in place of nucleic acid in the reaction mix, and a positive RNA amplification control consisting of combined wild-type and B.1.1.7 SARS-CoV-2 RNA obtained from clinical specimens positive by in-house endpoint PCR and sequencing. During optimization, it was determined that nonspecific signals in the N501Y target occurred above a *C_T_* of 37, as these were observed when making serial 10-fold dilutions of strongly positive (*C_T_* of <25) specimens known to be wild type based on Sanger sequencing. Due to this finding, and to provide consistent reporting guidelines, only results with a *C_T_* of ≤37 were considered positive for both probe targets. The assay does not have an indeterminate range. Any results with targets not detected (or *C_T_* of >37) were reported “unable to complete,” and if both N501 and N501Y were “unable to complete,” they would have an attached interpretation stating, “Unable to screen for N501Y gene mutations as SARS-CoV-2 virus was not detected with the multiplex VOC SNP assay.” An additional note would also be provided on the report stating, “SARS-CoV-2 VOC N501Y S gene mutation screening could not be performed as SARS-CoV-2 virus was not detected with the multiplex VOC SNP assay.” This could be due to low viral load in the specimen, PCR inhibition, or other technical issues. Note that the multiplex VOC SNP assay is less sensitive than SARS-CoV-2 PCR detection assays. The time interval between sequencing and running the N501Y rRT-PCR assay ranged from 1 to 14 days.

### PCR amplification and sequencing of partial spike gene.

Sanger sequencing was chosen as the comparator method, as it is a highly accurate methodology to determine the presence of SNPs and was the initial method used at PHO Laboratory for VOC detection. A detailed explanation of the Sanger sequencing protocol used is provided in Text S1. Specimens with *C_T_* values of ≤35 with a good quality Sanger chromatogram containing evenly spaced peaks each with one color and the least amount of baseline noise were considered positive. Sanger sequencing failed when *C_T_* values were greater than 35 and if the chromatogram contained mixed bases and was messy. We conducted Sanger sequencing directly from the specimen without additional cloning steps for specimens that had low viral load. GenBank accession numbers for our samples are OK413466 to OK413612.

### Validation.

Primary outcome measures were analytical sensitivity (95% limit of detection [LOD]), positive percent agreement (PPA), negative percent agreement (NPA), and precision.

A panel of 160 SARS-CoV-2-positive clinical specimens that had been characterized by partial Sanger sequencing of the S gene were used for validation of the N501Y SNP assay. The result obtained by our rRT-PCR N501Y SNP assay was compared to the known sequence results previously obtained by partial S gene sequencing and used to calculate PPA and NPA. PPA of >90% and NPA of >95% were considered acceptable. Different sets of specimens were used to conduct the precision experiments and LOD determination.

Analytical sensitivity (95% LOD) in copies per reaction was conducted for both N501Y and N501 targets in the N501Y SNP rRT-PCR. For the N501Y and N501 targets, we used a clinical specimen quantified using a known standard that is characterized as B.1.1.7 and wild type by WGS, respectively. Starting with 28.2 log copies/reaction of the B.1.1.7 specimen and 28.8 log copies/reaction of the wild-type specimen, 10-fold serial dilutions were performed down to 10^−10^ of the starting material in PCR-grade water and tested in our assay. The LOD experiments for both targets were done using 5 μL of nucleic acid and five replicates for each dilution. The LOD data were generated on three different days for a total of 15 replicates per dilution. The 95% LOD indicates the lowest concentration of genomic copies at which the target will be detected with a probability of 95%. A 95% LOD along with 95% confidence intervals were calculated using an online tool developed at PHO (available at https://biostats.shinyapps.io/LOD-new/).

Intrarun repeatability was evaluated for both N501Y and N501 targets starting with 6.18 log copies/reaction and 6.40 log copies/reaction, respectively, and 10-fold serial dilutions down to 10^−5^ of starting material were tested. We ran five replicates per dilution and compared *C_T_* values; we also report the percent agreement across the replicates for each dilution. The same analysis done for intrarun repeatability was done to assess interrun reproducibility for both N501Y and N501 targets; however, we conducted this analysis over 3 days. In total, we ran 15 replicates per dilution and compared *C_T_* values and reported the percent agreement across the replicates for each dilution. A panel of 10 N501Y VOCs (including one with a mixed population) and 5 wild-type specimens was sent out to six different microbiology laboratories in Ontario to provide interlaboratory comparison data and to assist those laboratories to validate the assay prior to implementation. The threshold for acceptance was greater than 95% agreement between labs. We compared *C_T_* values across the replicates and the percent agreement.

### Point prevalence and provincial implementation.

On 20 January 2021, all specimens that tested positive for SARS-CoV-2 were sent to PHO Laboratory for a point prevalence assessment of circulating VOCs. We received 2,756 specimens, but only 2,570 specimens were eligible for screening. Screening could not be completed for 186 specimens, likely due to low viral quantity. Specimens were screened using our N501Y RT-PCR assay to detect the presence of the N501Y mutation and were subsequently sequenced with WGS. Specimens underwent WGS as previously documented (https://doi.org/10.17504/protocols.io.bs98nh9w), and bioinformatics analysis was completed as referenced at https://github.com/jts/ncov2019-artic-nf.

### Lineage assignment.

The B.1.1.7 lineage was assigned via Sanger sequencing through the identification of mutations that occurred in the spike gene. We aligned our sequences to the B.1.1.7 reference. We looked at the following nucleotide positions in the S gene: G22813, G23012, and A23063, which correspond to K417, E484, and N501, respectively. If a sequence was wild type at positions 22813, 23012, and A23063T, which correspond to K417, E484, and N501Y, respectively, then it was called a probable UK variant. However, final lineage assignment was done after WGS. In the point prevalence study, lineage assignment was done through WGS.

### Statistical analysis.

Both the scatterplot and the Bland-Altman plots were created using the ggplot2 package in R 4.1.1. Simple linear regression and Pearson correlation (*r*) values were produced using R.

### Ethical statement.

The PHO Ethics Review Board has determined that this project did not require research ethics committee approval, as it describes analyses that were completed at PHO Laboratory as part of routine clinical respiratory testing and surveillance during the COVID-19 pandemic in Ontario and are, therefore, considered public health practice and are exempt from this requirement.

### Data availability.

All sequence data generated in this study are deposited at GenBank under accession numbers OK413466 to OK413612.
