# Supplementary material for: Real-Time RT-PCR Allelic Discrimination Assay for Detection of N501Y Mutation in the Spike Protein of SARS-CoV-2 Associated with B.1.1.7 Variant of Concern
Source: Microbiol Spectr. 2022 Feb 16;10(1):e00681-21. doi: 10.1128/spectrum.00681-21 (PMC8881086; doi:10.1128/spectrum.00681-21)
Supplement: SUPPLEMENTAL FILE 1 — Supplemental material. Download SPECTRUM00681-21_Supp_1_seq6.pdf, PDF file, 0.2 MB [file spectrum00681-21_supp_1_seq6.pdf]

Supplementary Table 1: Primers and probes used in the PHO Laboratory SARS-CoV-2 N501Y SNP rRT-PCR assay and Sanger sequencing

Name	Sequence (5'-3')	Position	Final Concentration (nM)
**SNP assay primers and probes**			
501F	GAAGGTTTTAATTGTTACTTTC	23012-23033	1000
501R	AAACAGTTGCTGGTGCATGT	23116-23136	1000
FAM-Y501 (T23063)	CCAACCCACT*T*ATGGTGTTG	23053-23072	250
HEX-N501 (A23063)	CCAACCCACT*A*ATGGTGTTG		500
**S gene fragment RT-PCR and sequencing primers**			
VOCF	AGAGTCCAACCAACAGAATCTATTGT	22517-22542	400

VOCR	ACACCTGTGCCTGTTAAACCAT	23192-23214	400

Supplementary Table 2: Summary of intra-run repeatability for N501Y target

Dilution	Mean N501Y Ct	S.D[b]	CV (%)[c]	Positive (%)
UD[a]	18.18	0.07	0.40	5 (100%)
10^{-1}	21.49	0.14	0.66	5 (100%)
10^{-2}	24.98	0.08	0.31	5 (100%)
10^{-3}	28.41	0.17	0.60	5 (100%)
10^{-4}	31.63	0.15	0.48	5 (100%)
10^{-5}	35.29	0.62	1.75	5 (100%)

a. UD= Undiluted
b. SD= Standard deviation
c. CV= Coefficient of variation

Supplementary Table 3: Summary of intra-run repeatability for N501 target

Dilution	Mean N501 Ct	SD[b]	CV (%)[c]	Positive (%)
UD[a]	18.22	0.18	0.97	5 (100%)
10^{-1}	21.46	0.13	0.61	5 (100%)
10^{-2}	24.88	0.18	0.71	5 (100%)
10^{-3}	28.44	0.16	0.58	5 (100%)
10^{-4}	31.63	0.22	0.71	5 (100%)
10^{-5}	35.03	0.30	0.87	5 (100%)

a. UD= Undiluted
b. SD= Standard deviation
c. CV= Coefficient of variation

Supplementary Table 4: Summary of inter-run laboratory reproducibility for N501Y target

Dilution	Mean N501Y Ct Day 1	Mean N501Y Ct Day 2	Mean N501Y Ct Day 3	Overall Mean N501Y Ct	S.D[b]	CV[c] (%)	Positive (%)
UD[a]	18.18	18.30	18.35	18.28	0.17	0.92	15 (100%)
10^{-1}	21.49	21.43	21.63	21.51	0.17	0.77	15 (100%)
10^{-2}	24.98	24.92	25.94	25.28	0.91	3.61	15 (100%)
10^{-3}	28.41	28.37	28.51	28.43	0.15	0.54	15 (100%)
10^{-4}	31.63	31.68	32.01	31.77	0.23	0.71	15 (100%)
10^{-5}	35.29	34.87	35.22	35.13	0.54	1.53	15 (100%)

a. UD= Undiluted
b. SD= Standard Deviation
c. CV= Coefficient of Variation

Supplementary Table 5: Summary of inter-run reproducibility for N501 target

Dilution	Mean N501 Ct Day 1	Mean N501 Ct Day 2	Mean N501 Ct Day 3	Overall Mean N501 Ct	SD[b]	CV[c] (%)	Positive (%)
UD[a]	18.22	17.06	18.32	17.87	0.71	4.00	15 (100%)
10^{-1}	21.46	20.42	21.47	21.12	0.60	2.84	5 (100%)
10^{-2}	24.88	23.87	24.80	24.52	0.57	2.33	5 (100%)
10^{-3}	28.44	27.46	28.33	28.07	0.54	1.94	5 (100%)
10^{-4}	31.63	30.58	31.69	31.30	0.62	1.97	5 (100%)
10^{-5}	35.03	33.96	35.81	34.93	1.03	2.95	5 (100%)

a. UD= undiluted
b. S.D= Standard Deviation
c. CV= Coefficient of Variation

Supplementary Table 6: Summary of inter-laboratory reproducibility for N501Y target

Sample	Mean N501Y Ct from Partnering Laboratories (Replicates=6))	S.D[a]	CV (%)[b]	Positive (%)	Mean N501Y Ct from PHO Laboratory (Replicates=4)	S.D[a]	CV (%)[b]	Positive (%)
1	21.41	0.86	4.03	6 (100%)	20.36	0.4	2.14	4 (100%)
2	31.28	1.05	3.34	6 (100%)	29.65	0.38	1.27	4 (100%)
3	26.46	0.72	2.70	6 (100%)	25.15	0.38	1.50	4 (100%)
4	32.32	1.12	3.48	6 (100%)	30.52	0.48	1.57	4 (100%)
5	24.40	2.21	9.05	6 (100%)	21.81	0.58	2.66	4 (100%)
6	24.56	0.78	3.17	6 (100%)	23.19	0.36	1.54	4 (100%)
7	30.60	1.17	3.83	6 (100%)	29.07	0.36	1.24	4 (100%)
8	25.42	1.03	4.06	6 (100%)	24.37	0.32	1.33	4 (100%)
9	33.35	2.68	8.03	6 (100%)	30.82	1.06	3.43	4 (100%)
10	30.43	0.88	2.88	6 (100%)	29.38	0.44	1.51	4 (100%)

a. S.D= Standard Deviation
b. CV= Coefficient of Variation

Supplementary Table 7: Summary of inter-laboratory reproducibility for N501 target

Sample	Mean N501 Ct from Partnering Laboratories (Replicates=6))	S.D[a]	CV (%)[b]	Positive (%)	Mean N501 Ct from PHO Laboratory (Replicates=4)	S.D[a]	CV (%)[b]	Positive (%)
1	29.04	1.16	4.00	6 (100%)	28.51	0.4	1.54	4 (100%)
2	23.31	1.15	4.93	6 (100%)	23.26	0.46	1.97	4 (100%)
3	23.81	1.04	4.35	6 (100%)	23.47	0.45	1.93	4 (100%)
4	24.36	1.36	5.59	6 (100%)	24.09	0.31	1.28	4 (100%)
5	30.36	1.27	4.17	6 (100%)	29.82	0.36	1.20	4 (100%)
6	29.74	1.69	5.70	6 (100%)	30.18	0.25	0.84	4 (100%)

a. S.D= Standard Deviation
b. CV= Coefficient of Variation

Supplementary Methods:

PCR Amplification and Sequencing of Partial Spike Gene Methodology:
Primers were designed to amplify a 698 bp S gene fragment spanning the region of interest (genome positions: 22516 to 23214). Reverse transcription and amplification of the partial S gene was performed using the OneStep RT-PCR kit (QIAGEN, Hilden, Germany). A 25 µl reaction mixture contained 5 µl of 5X QIAGENOneStep RT-PCR Mix, 1 µl (200 µM) of dNTPs, 1 µl of OneStep RT-PCR enzyme mix, 1 µl of each forward (VOCF) and reverse (VOCR) primers at a final concentration of 0.4 µM (**Supplementary Table 1**), 11µl of water and 5µl of RNA. Reactions were carried out on the Applied Biosystems™ SimpliAmp® Thermal Cycler (Thermo Fisher Scientific, Massachusetts, United States) with the following conditions: 1 cycle at 50 °C for 30 minutes and 1 cycle at 95 °C for 15 minutes, followed by 40 cycles of

amplification at 94 °C for 30 seconds, 56 °C for 30 seconds, and 72 °C for 1 minute, and a final extension cycle of 72 °C for 10 minutes.

Applied Biosystems™ BigDye™ Terminator v3.1 Cycle Sequencing Kit (Thermo Fisher Scientific, Massachusetts, United States) was used for bidirectional sequencing of PCR fragments following manufacturer's recommendations. Each reaction contained 0.5 µl of Terminator Ready Reaction Mix, 3.75 µl of 5X buffer, 1µl of primer mix and 1 µl amplified PCR product, and 13.75µl of water in a final volume of 20 µl. Default cycling conditions for the BigDye™ Terminator reaction were used as follows: 96°C for 1 minute followed by 35 cycles of 96°C for 10 seconds, 50°C for 5 seconds, and 60°C for 4 minutes. BigDye™ reaction products were purified using an EDTA/ ethanol precipitation method and resuspended in 20µl Hi-Di™ Formamide (Thermo Fisher Scientific, Massachusetts, United States). Nucleotide sequences were determined using the Applied Biosystems™ ABI 3130xl Genetic Analyzer (Thermo Fisher Scientific, Massachusetts, United States) and analyzed using FinchTV software (Geospiza, Inc.).
